# State level variations in nephrology workforce and timing and incidence of dialysis in the United States among children and adults: a retrospective cohort study

**DOI:** 10.1186/1471-2369-16-2

**Published:** 2015-01-15

**Authors:** Elaine Ku, Kirsten L Johansen, Anthony A Portale, Barbara Grimes, Chi-yuan Hsu

**Affiliations:** Division of Nephrology, Department of Medicine, University of California, San Francisco, 521 Parnassus Avenue, C443, Box 0532, San Francisco, CA USA; Department of Pediatrics, Division of Pediatric Nephrology, 533 Parnassus Ave, UC Hall Box 0748, San Francisco, CA 94143 USA; Department of Biostatistics and Epidemiology, 550 16th Street, 2nd Floor, Box 0560, San Francisco, CA 94158 USA; United States Renal Data System, Nutrition Special Studies Center, University of California, San Francisco, 521 Parnassus Avenue, C443, Box 0532, San Francisco, CA 94143-0532 USA

**Keywords:** Nephrology workforce, Timing of dialysis initiation

## Abstract

**Background:**

Multiple factors influence timing of dialysis initiation. The impact of supply of nephrology workforce on timing and incidence of dialysis initiation is not well known.

**Methods:**

We determined the number of pediatric and adult nephrologists in each state using data from the American Medical Association and American Boards of Internal Medicine and Pediatrics. We ascertained state population data from the 2010 US Census. United States Renal Data System (USRDS) data were used to determine estimated glomerular filtration rate (eGFR) at dialysis initiation and dialysis incidence for adults (≥18 years) in 2008 and children (<18 years) in 2007–2009 by state.

**Results:**

Across all states, there were a median of 3.0 (IQR 2.3 to 3.4) adult nephrologists per 100,000 adults and 0.5 (IQR 0.2 to 0.9) pediatric nephrologists per 100,000 children. The median eGFR at start of dialysis was 9.8 mL/min/1.73 m^2^ (IQR 7.1-13.1) in adults and 8.5 mL/min/1.73 m^2^ (IQR 6.2-11.4) in children. Neither the number of adult (Spearman r of 0.02 [95% CI −0.26-0.30], p = 0.88) nor pediatric (Spearman r of −0.13 [95% -0.39-0.15], p = 0.38) nephrologists per state population was associated with mean eGFR across states. The number of nephrologists per state population was associated with incident dialysis cases per state population in adults (Spearman r of 0.50 [95% CI 0.26-0.68], p = 0.0002), but not in children (Spearman r of −0.06 [95% CI −0.33-0.22], p = 0.67). In linear regression models, the association between nephrologists per state population and incident dialysis cases per state population remained statistically significant (p = 0.006) after adjustment for provider characteristics.

**Conclusions:**

Nephrology workforce supply is aligned with demand but does not appear to be associated with timing of dialysis initiation.

## Background

A number of clinical and socioeconomic factors influence the timing of dialysis initiation. Little is known about the impact of the nephrology workforce supply.

A recent trend towards earlier dialysis initiation at higher estimated glomerular filtration rates (eGFR) has been noted in both adult and pediatric nephrology [1–3]. The cause of this shift in practice is unclear. Various studies have cited frailty, advanced age, presence of multiple comorbidities, and discrepancies between measured and estimated GFR in sarcopenic patients as potential factors explaining the trend towards earlier dialysis initiation [4–7]. Regardless of the etiology, the shift in practice towards earlier dialysis initiation is concerning given that one large randomized controlled trial and several observational studies have failed to demonstrate any mortality benefit with earlier dialysis initiation [1, 8, 9]. Earlier dialysis initiation also has not been shown to improve quality of life or the cost-effectiveness of care [10]. In a recent survey of European nephrologists, opinions regarding the optimal timing of dialysis initiation differed based on whether respondents were from for-profit versus non-profit centers; those from for-profit organizations were more likely to favor early dialysis initiation [11]. In contrast, a Canadian study found that patient characteristics, as opposed to dialysis facility or geographic factors, contributed the most to the explained variation in eGFR at dialysis initiation [12].

Few studies have examined whether the density of nephrologists is associated with timing of dialysis initiation or dialysis incidence. In subspecialties other than nephrology, physician supply has been shown to influence healthcare consumption via a “supplier-induced demand” [13, 14]. For example, in one Canadian study, the density of physicians was more strongly associated with use of cardiovascular testing than was disease burden [15].

In the present study, we examined the association of US nephrologist supply with the timing and rate of dialysis initiation by state. We hypothesized that dialysis would be initiated at a higher eGFR in states with more nephrologists per capita and that dialysis incidence would be higher in states with a higher number of nephrologists per capita.

## Methods

### Determination of state level variation in nephrology workforce

To estimate the size and location of the current nephrology workforce, we used deidentified data from the 2008 American Medical Association (AMA) Physician Professional Data (PPD) Statistical Research file, which contains data on the primary specialty, demographic characteristics, type of practice (private versus academic versus research), and location of physicians in the US [16, 17]. The state of practitioners’ office address was used to determine current location of nephrologists, but if office addresses were not available, the state of the practitioner’s preferred mailing address was used. Physicians known to be retired, deceased, in training programs through the end of 2008, have undeliverable addresses, or missing location per AMA-PPD database were excluded.

In sensitivity analysis, we confirmed the size and location of the current nephrology workforce derived from the AMA-PPD file using most recent data available at time of study performance from the American Boards of Internal Medicine (ABIM) and Pediatrics (ABP), which were updated as of 2/17/2011 and 12/31/2010, respectively. Both organizations publish the total number of board-certified adult and pediatric nephrologists annually and use updated candidate addresses on file to assign their locations by state. In the ABIM database, certified subspecialists known to be deceased are excluded [18]. In the ABP database, candidates with missing age data or candidates known to be 66 years of age or older are presumed to be retired and excluded [19]. State population data were derived from the 2010 United States Census. The number of adult and pediatric nephrologists was calculated per 100,000 adults or children, respectively, in each of the fifty states, with exclusion of the District of Columbia.

### Determination of state level estimated glomerular filtration rate at dialysis initiation and dialysis incidence

The United States Renal Data System (USRDS) is a national registry that tracks epidemiology and outcomes data on patients with end-stage renal disease (ESRD). We identified all adult patients initiated on hemodialysis and peritoneal dialysis in 2008 and all pediatric patients initiated on hemodialysis and peritoneal dialysis between 2007–2009 in each state based on the most recent data available at time this study was initiated. We included three years of pediatric data in order to minimize random variation by state, as small numbers of pediatric patients initiate dialysis each year. We selected 2008 for adult data analysis since 2008 lies within the time span of the pediatric data analysis. Data on age, sex, race (African American versus other), Medicaid status (yes/no as a measure of lower income status), cause of ESRD (diabetes versus other), presence of congestive heart failure (CHF), serum creatinine, serum albumin, serum hemoglobin, and height for children were extracted from the End-stage Renal Disease Medical Evidence Report (CMS-2728) submitted around the time of outpatient dialysis initiation (USRDS MEDEVID file). We stratified state-level patient characteristics at incident ESRD by the lowest and highest quintiles of nephrology workforce to determine the presence of any potential differences.

Estimated GFR at the start of dialysis was determined for adults (≥18 years) using the Modified Diet in Renal Disease (GFR = 175 × serum creatinine^−1.154^ × age^−0.203^ × 1.212 [if black] × 0.742 [if female]) [20] and for children (<18 years) using the updated Schwartz ([0.41 × height]/serum creatinine) [21] equations. If eGFR was < 2 mL/min/1.73 m^2^ or > 25 mL/min/1.73 m^2^, eGFR was set to 2 mL/min/1.73 m^2^ or 25 mL/min/1.73 m^2^ respectively for purposes of statistical analysis in both adult and pediatric data, and results were averaged for each state.

For the eGFR analysis, patients who lacked sufficient data to estimate GFR were excluded from analysis. This included 470 adults due to missing serum creatinine and 225 children due to missing height (n = 173), serum creatinine (n = 34), or both (n = 18), although these patients were still included in the analyses of dialysis incidence.

### Provider characteristics

Using data from the AMA-PPD file, we determined the age of current adult and pediatric nephrologists as of 2008, sex, years since graduation from training (> versus ≤ 10 years), location of medical training (US versus foreign), degree type (Doctor of Medicine (MD) versus Doctor of Osteopathic Medicine (DO)), primary role (direct patient care versus other), and primary employment (private/group practice or health care maintenance organization (HMO) versus medical school versus other).

### State population characteristics

To assess patient-related characteristics, we determined the percentage of the adult population with obesity (defined as body mass index ≥ 30) in 2008 and with diabetes in 2010 by state, using data from the Center for Disease Control and Prevention [22, 23]. For children, we determined the percentage of obesity (defined as ≥ 95^th^ percentile) in 2007 by state according to data from the National Survey of Children’s Health [24]. We also determined the percentage of the total population in each state 65 years of age or older, African American, or living in rural (versus urban) areas according to the 2010 US census [25–27].

We stratified state-level population characteristics by the highest and lowest quintiles of nephrology workforce to determine potential differences between states with the highest and lowest density of nephrology workforce.

### Statistical methods

All analyses were conducted at the level of the state. Spearman coefficients were determined for the association between the number of adult or pediatric nephrologists per 100,000 population in each state and the mean eGFR at start of dialysis and dialysis incidence in each state per 100,000 population.

We then used multivariate linear regression models to further understand the association between density of nephrology workforce (independent variable) and mean eGFR or number of incident dialysis cases (dependent variables) for adults and children separately in each state. Specifically, we examined the association between density of nephrology workforce and outcome after adjusting first for provider characteristics (age, sex, years since training, US versus foreign-trained, MD versus DO, direct patient care [yes/no], and primary employment [private practice versus medical school versus other]) at the state level for adults and children. Subsequently, in separate multivariate linear regression models, we adjusted for state population characteristics (percent diabetes, percent obesity, and percent age ≥ 65 years in adults; percent obesity in children; percent African American and percent rural population [based on total state population estimates] in both adults and children). In a third linear regression model, we adjusted for patient-level characteristics measured at the state level at time of dialysis initiation, including percentage of African Americans, percentage with diabetes as cause of ESRD, percentage with CHF, Medicaid status, mean serum hemoglobin, mean serum albumin, and percentage with age ≥ 65 (in adult model only).

In sensitivity analysis, we approximated “full time equivalency” in our workforce density by multiplying the percentage of providers reporting direct patient care as their primary role in each state with our state-level workforce density and repeated our analyses (both Spearman coefficients and all linear regression models) using these attenuated workforce densities for adults and children. We also excluded states without transplants in adults (Alaska, Idaho, Montana, Wyoming) and children between 2007–2009 (Alaska, Idaho, Kansas, Montana, Wyoming) according to the Organ Procurement and Transplantation Network and repeated our Spearman correlations using this subgroup [28].

The Committee on Human Research of the University of California San Francisco consider the scope of this work to be exempt human subjects research.

## Results

105,178 adults initiated dialysis in 2008, and 2,136 children initiated dialysis between January 1, 2007 and December 31, 2009 in the US. Mean eGFR at the start of dialysis was 10.6 mL/min/1.73 m^2^ (median 9.8, IQR 7.1-13.1) in adults and 9.5 mL/min/1.73 m^2^ (median 8.5, IQR 6.2-11.4) in children across the US.

At the state level, the median number of adult nephrologists was 3.0 (IQR 2.3 to 3.4) per 100,000 adults, and the median number of pediatric nephrologists was 0.5 (IQR 0.2 to 0.9) per 100,000 children (Table 1) using data from the AMA-PPD. In sensitivity analysis using data from the American Boards of Internal Medicine and Pediatrics, the median number of adult nephrologists was 3.2 (IQR 2.6 to 3.8) per 100,000 adults, and the median number of pediatric nephrologists was 0.5 (IQR 0.3 to 0.7) per 100,000 children across states. Accounting for the percentage of providers reporting direct patient care as their primary role in each state, the median number of adult nephrologists was 2.5 (IQR 2.0 to 2.9) per 100,000 adults per AMA-PPD database, and the median number of pediatric nephrologists was 0.4 (IQR 0.2 to 0.5) per 100,000 children per AMA-PPD database.

**Table 1:** **Workforce by state based on data available through the American Medical Association in 2008**

State	Number of adult nephrologists (N = 7607)	Number of pediatric nephrologists (N = 509)	Number of adult nephrologists/100,000 adults	Number of pediatric nephrologists/100,000 children
Alabama	109	3	3	0.3
Alaska	4	1	0.8	0.5
Arizona	137	3	2.9	0.2
Arkansas	50	4	2.3	0.6
California	862	60	3.1	0.6
Colorado	98	3	2.6	0.2
Connecticut	127	9	4.6	1.1
Delaware	28	2	4	1
Florida	443	30	3	0.7
Georgia	235	7	3.3	0.3
Hawaii	23	1	2.2	0.3
Idaho	18	0	1.6	0
Illinois	297	27	3.1	0.9
Indiana	155	4	3.2	0.2
Iowa	47	1	2	0.1
Kansas	55	2	2.6	0.3
Kentucky	94	7	2.8	0.7
Louisiana	137	5	4	0.4
Maine	26	4	2.5	1.5
Maryland	193	11	4.4	0.2
Massachusetts	270	25	5.3	1.8
Michigan	244	15	3.2	0.6
Minnesota	139	15	3.5	1.2
Mississippi	69	1	3.1	0.1
Missouri	144	14	3.2	1
Montana	8	0	1	0
Nebraska	32	2	2.3	0.4
Nevada	46	2	2.3	0.3
New Hampshire	19	0	1.8	0
New Jersey	271	11	4	0.5
New Mexico	32	3	2.1	0.6
New York	703	53	4.7	1.2
North Carolina	245	15	3.4	0.7
North Dakota	14	0	2.7	0
Ohio	266	29	3	1.1
Oklahoma	61	1	2.2	0.1
Oregon	65	5	2.2	0.6
Pennsylvania	395	25	4	0.9
Rhode Island	35	3	4.2	1.3
South Carolina	109	6	3.1	0.6
South Dakota	22	0	3.6	0
Tennessee	167	14	3.4	0.9
Texas	597	43	3.3	0.6
Utah	33	3	1.7	0.3
Vermont	10	0	2	0
Virginia	182	12	3	0.6
Washington	138	16	2.7	1
West Virginia	40	2	2.7	0.5
Wisconsin	109	10	2.5	0.7
Wyoming	4	0	0.9	0

Across states, 78.4% of adult nephrologists were male, 45.8% finished training within the last 10 years, and 62.0% were currently employed by private practice or HMO (Table 2). In contrast, across states, 57.5% of pediatric providers were male, 48.5% finished training more than 10 years ago, and 38.8% were employed in private practice or HMO.

**Table 2:** **Characteristics of adult and pediatric nephrologists based on AMA-PPD file averaged across states**

Characteristic of nephrologists	Adult (all states)	Pediatric* (all states)
**Median age, years**	48.3	48.0
[25^th^-75^th^ percentile]	[47.4-49.5]	[45.9-49.5]
**Male** (%)	78.4	57.5
**US trained** (%)	36.6	34.1
**Direct patient care** (%)	85.7	68.1
**Doctor of Osteopathy** (%)	3.6	1.6
**Within 10 years graduation from training** (%)	45.8	48.5
**Private practice** (%)	62.0	38.8

*Averaged across states with pediatric nephrologists (N = 43).

At the state level, mean prevalence of obesity was 26.7% in adults and 15.2% in children, mean prevalence of African Americans across states was 11.3%, and on average 26.4% of state populations were rural. In adults, the mean state prevalence of diabetes was 8.2% and the mean state prevalence of populations 65 years of age or older was 13.3%. No significant differences were noted in state-level population characteristics between the highest and lowest quintiles of workforce density, except that states with the highest quintile of workforce density had significantly lower percentage of the population living in rural areas and higher percentage of African Americans compared to states with the lowest quintile of workforce density (Tables 3 and 4).

**Table 3:** **Characteristics of the adult population by lowest and highest quintiles of adult nephrology workforce density based on AMA-PPD file**

Characteristic	Average % or value amongst states in lowest quintile of adult nephrology workforce density (± SD)	Average % or value amongst states in highest quintile of adult nephrology workforce density (± SD)	P-value ^1^
**State population characteristics**			
Obesity	25.7 ± 2.3	25.4 ± 3.0	0.97
Age ≥ 65 years*	12.6 ± 2.4	13.8 ± 0.96	0.40
African American*	2.8 ± 2.4	15.9 ± 10.2	<0.001
Rural population*	34.5 ± 13.4	16.8 ± 11.3	0.008
Diabetes	7.2 ± 1.2	7.9 ± 1.2	0.12
**Incident ESRD population characteristics**			
Age ≥ 65 years	51.2 ± 5.7	54.1 ± 5.9	0.45
African American	4.7 ± 4.6	28.2 ± 18.5	0.004
Diabetes as cause of ESRD	45.8 ± 8.1	41.3 ± 4.3	0.26
Congestive heart failure	28.1 ± 8.1	36.8 ± 4.8	0.02
Medicaid	18.9 ± 6.2	22.7 ± 6.3	0.21
Mean hemoglobin (g/dL)	10.2 ± 0.1	9.9 ± 0.1	<0.001
Mean albumin (g/dL)	3.2 ± 0.1	3.1 ± 0.1	0.03

^1^Wilcoxin rank sum test.

*Percentages based on total state population.

**Table 4:** **Characteristics of the pediatric population by lowest and highest quintiles of pediatric nephrology workforce density based on AMA-PPD file**

Characteristic	Average % or value amongst states in lowest quintile of pediatric nephrology workforce density (± SD)	Average % or value amongst states in highest quintile of pediatric nephrology workforce density (± SD)	P-value ^1^
**State population characteristics**			
Obesity	13.4 ± 3.4	13.8 ± 2.4	0.31
African American*	6.0 ± 11.4	10.5 ± 6.3	0.02
Rural population*	41.3 ± 9.2	21.4 ± 15.8	0.003
**Incident ESRD population characteristics**			
African American	9.5 ± 23.4	10.4 ± 10.8	0.15
Diabetes as cause of ESRD	1.0 ± 2.3	2.2 ± 3.6	0.35
Congestive heart failure	4.2 ± 10.6	1.3 ± 1.8	0.38
Medicaid	50.9 ± 28.4	47.8 ± 24.4	0.59
Mean hemoglobin (g/dL)	10.3 ± 1.1	9.7 ± 0.7	0.23
Mean albumin (g/dL)	3.5 ± 0.5	3.2 ± 0.5	0.22

^1^Wilcoxin rank sum test.

*Percentages based on total state population.

Patient characteristics at time of incident ESRD averaged across states are shown in Tables 3 and 4. There was a statistically significantly lower mean hemoglobin and mean albumin at ESRD onset in states with the highest nephrologist density in adults, but not in children, although these differences were not clinically significantly different. A significantly higher mean prevalence of African Americans was noted in incident ESRD patients in states with the highest workforce density in adults (Table 3).

**Figure 1:**
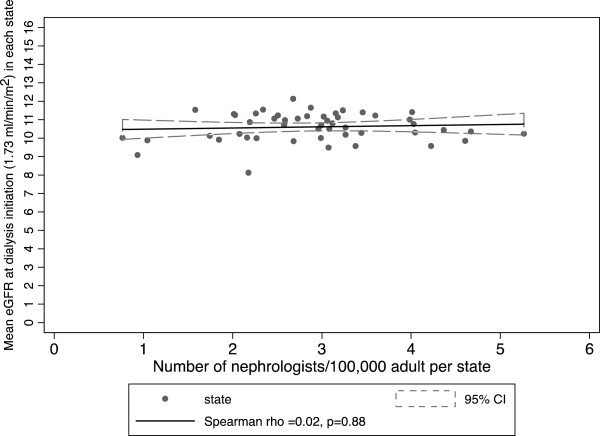
**Mean eGFR at dialysis initiation among adult patients versus number of adult nephrologists per 100,000 adult population by state.**

**Figure 2:**
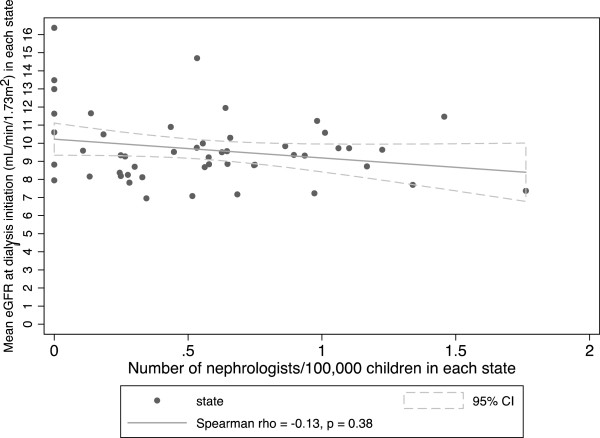
**Mean eGFR at dialysis initiation among children versus number of pediatric nephrologists per 100,000 children by state.**

The mean eGFR at the start of dialysis did not correlate with the number of nephrologists per state population in adults (Spearman r of 0.02 [95% CI −0.26-0.30], p = 0.88) (Figure 1) or children (Spearman r of −0.13 [95% CI −0.39-0.15], p = 0.38) (Figure 2). In multivariable linear regression models, there was no statistically significant association between the number of nephrologists per state population and mean eGFR in adults or children. This is true even with adjustment for patient, provider, and population characteristics at the state level (Table 5).

**Table 5:** **Adjusted linear regression models of the association between nephrologist workforce density and mean eGFR by state**

Linear regression model for mean eGFR	Adult	95% CI	Children	95% CI
	Change in mean eGFR per 1 unit increase in workforce density*		Change in mean eGFR per 1 unit increase in workforce density*	
Unadjusted	0.06	[−0.17, 0.30]	−1.03	[−2.40, 0.32]
Adjusted for provider characteristics^1^	−0.15	[−0.35, 0.05]	0.32	[−1.14, 1.78]
Adjusted for state population characteristics^2^	0.27	[−0.10, 0.64]	−0.62	[−1.70, 0.46]
Adjusted for patient characteristics at incident ESRD^3^	0.13	[−0.23, 0.48]	−0.67	[−1.94, 0.60]

^1^Adjusted for age of providers in 2008, sex, US versus foreign medical graduate, DO versus MD, > or ≤ 10 years since training, primary role (direct patient care versus other), and present employment (private practice or HMO versus other); N = 43 states for pediatric analyses with exclusion of 7 states without providers where provider characteristics could not be determined.

^2^Adjusted for percentage of state population with obesity, African American, diabetes, ≥ 65 years of age, rural population in adults and percentage obesity, African American, and rural population in children.

^3^Adjusted for race (African American versus other), diabetes as cause of ESRD (yes/no), CHF (yes/no), Medicaid status (yes/no), mean hemoglobin, mean albumin, and age ≥ 65 years (in adult model only); N = 49 states for pediatric analysis due to missing comorbidity and demographic data on the 1 ESRD case in Wyoming.

*Per 1 additional nephrologist/100,000.

**Figure 3:**
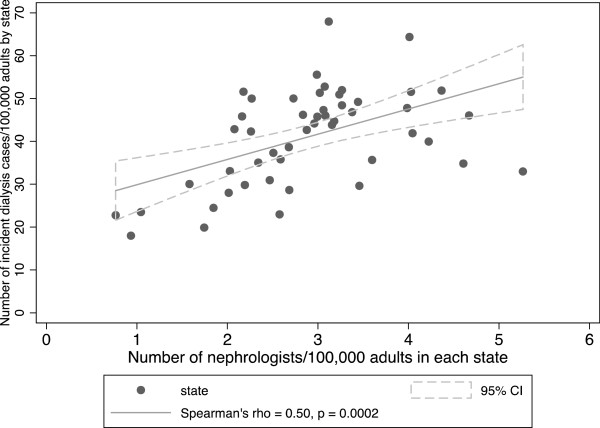
**Dialysis incidence among adult patients vs. number of nephrologists per 100,000 adults by state.**

**Figure 4:**
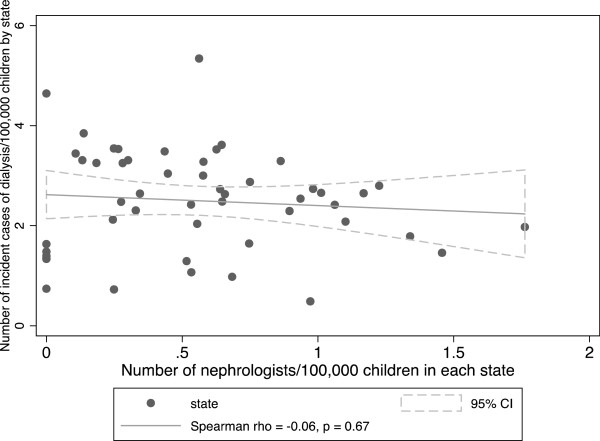
**Dialysis incidence among pediatric patients vs. number of nephrologists per 100,000 children by state.**

Among adults, the mean incidence of dialysis per 100,000 state population was 40.9 (median 43.2, IQR 33.0-49.0). Among children, the mean incidence of dialysis per 100,000 children in each state was 2.5 (median 2.6, IQR 1.8-3.2). We observed a statistically significant association between incident cases of dialysis per state and the number of nephrologists/state population in adults (Spearman r of 0.50 [95% CI 0.26-0.68], p = 0.0002) (Figure 3), but not in children (Spearman r of −0.06 [95% CI −0.33-0.22], p = 0.67) (Figure 4).

In linear regression models adjusted for provider characteristics at the state level, there was a statistically significant association between number of nephrologists per state population and number of incident dialysis cases per state population in adults. Every additional adult nephrologist per 100,000 population was associated with 5.88 more incident cases of ESRD per 100,000 adults per state. This estimate was largely unchanged after adjustment for provider characteristics but was no longer evident after adjusting for either state-level population characteristics or state-level patient characteristics at incident ESRD (Table 6).

**Table 6:** **Adjusted linear regression models of the association between nephrologist workforce density and incident number of ESRD cases per 100,000 population by state**

Linear regression model for number of cases of ESRD/100,000 population	Adult	95% CI	Children	95% CI
	Change in incident ESRD cases/100,000 population per 1 unit increase in workforce density ^*^		Change in incident ESRD cases/100,000 population per 1 unit increase in workforce density ^*^	
Unadjusted	5.88	[2.30, 9.47]	−0.22	[−0.86, 0.42]
Adjusted for provider characteristics^1^	5.32	[1.63, 9.01]	−0.80	[−1.41, −0.18]
Adjusted for state population characteristics^2^	1.21	[−1.45, 3.86]	−0.35	[−0.86, 0.16]
Adjusted for patient characteristics at incident ESRD^3^	−0.70	[−3.43, 2.03]	−0.34	[−0.93, 0.25]

^1^Adjusted for age of providers in 2008, sex, US versus foreign medical graduate, DO versus MD, > or ≤ 10 years since training, primary role (direct patient care versus other), and present employment (private practice or HMO versus other); N = 43 states for pediatric analyses with exclusion of 7 states without providers where provider characteristics could not be determined.

^2^Adjusted for percentage of state population with obesity, African American, diabetes, ≥ 65 years of age, rural population in adults and percentage obesity, African American, and rural population in children.

^3^Adjusted for race (African American versus other), diabetes as cause of ESRD (yes/no), CHF (yes/no), Medicaid status (yes/no), mean hemoglobin, mean albumin, and age ≥ 65 years (in adult model only); N = 49 states for pediatric analysis due to missing comorbidity and demographic data on the 1 ESRD case in Wyoming.

*Per 1 additional nephrologist/100,000.

In children, none of the models showed positive correlation between number of pediatric nephrologists per state population and pediatric dialysis incident rate.

In sensitivity analysis using attenuated workforce densities that account for the percentage of providers in each state reporting direct patient care as their primary role, no major differences were noted in results by Spearman correlation or linear regression models for adults or children using outcomes of mean eGFR or incident ESRD cases per state population (results not shown). We also repeated Spearman correlations using only states with mean eGFR > 10 mL/min/1.73 m^2^ at dialysis initiation and found similar results (not shown). Excluding states without transplants between 2007–2009, we repeated Spearman correlations and found similar results in adults (results not shown) and children.

## Discussion

In this study, we examined the association between state-level variations in the supply of nephrologists and the timing and incidence of dialysis initiation across the United States. Our data do not support the hypothesis that variations in physician density contribute to earlier dialysis initiation in the US. Specifically, we found no significant association between the number of nephrologists per capita and the mean eGFR at start of dialysis in either the adult or pediatric population. Even in states with a relative abundance of nephrologists, we found no trend towards earlier dialysis initiation, although the incidence of dialysis initiation is higher in the adult population of these states in our primary analyses. The correlation between number of adult nephrologists per state population and incident adult dialysis cases per state was confirmed using linear regression models, even after adjustment for provider characteristics.

The relationship between physician decision-making and non-clinical or financial incentives is often complex, inconsistent, and difficult to model [29]. For example, in oncology, although financial incentives do not influence the decision to administer chemotherapy for cancer treatment, they do appear to influence the selection of chemotherapy agents [30]. In nephrology, recent increases in reimbursement rates for peritoneal dialysis relative to other renal replacement modalities have not resulted in a parallel increase in peritoneal dialysis use in either Canada or Germany [31]. It is likely that our model of workforce supply is somewhat simplified, as we are only able to adjust crudely for potential confounding factors such as insurance payor and modality of physician reimbursement (private practice or HMO versus other types of employment). Thus, we may have been unable to capture an association between nephrologist supply and timing of dialysis initiation, even if such a relation existed.

On the other hand, the complexity of medical management of patients with advanced chronic kidney disease may diminish the potential influence of physician density on mean eGFR at dialysis initiation. In the outpatient setting, management of chronic kidney disease complications such as fluid overload, electrolyte disturbances, anemia, and hypertension in patients with low eGFR requires frequent and close monitoring, which may lead to significant provider and patient burden. Initiation of dialysis may facilitate the management of these various complications of kidney disease and be seen by providers as an option that lessens their workload while enhancing the safety of patients [32]. Thus, mean eGFR may be relatively high even in areas with low nephrologist density where demand for nephrology services may preclude the time investment required for the complex medical outpatient management of patients with very low eGFR not on dialysis.

Regarding the pediatric nephrology workforce, we had hypothesized that states with lower physician density would have lower mean eGFR at time of dialysis initiation. Several states have no pediatric nephrologists (Table 1), and our hypothesis predicts that dialysis initiation would occur at lower levels of GFR in those states because of delayed access to care. However, we found no clear association between nephrologist density and mean eGFR at time of dialysis initiation, even in linear regression models adjusted for provider, population, and patient characteristics at the state level. The lack of association between pediatric physician density per capita and mean eGFR at dialysis initiation in our study suggests that workforce supply limitations have not led to late dialysis initiation in the US, at least as determined by eGFR.

Several factors may limit our ability to detect an association between physician density and mean eGFR at dialysis initiation in children, even if such a relation existed. In children, growth failure and certain rare genetic conditions such as primary hyperoxaluria can compel dialysis initiation at higher eGFRs, which may skew the mean eGFR, although we have used Spearman correlations throughout our study to model non-parametric distributions in our data. Adult practitioners often care for adolescents in areas where pediatric nephrology care is unavailable, but we cannot estimate the number of such practitioners when estimating the size of the pediatric workforce. Our analysis may lack sufficient power to detect a significant correlation between pediatric workforce density and dialysis initiation, given the relatively low number of children initiated on dialysis annually and missing data in USRDS, which prevented eGFR computation in approximately ten percent of the pediatric study population. For example, the seven states with no pediatric nephrologists only accounted for 26 cases of dialysis initiation over the three-year time span of our study.

We did find a significant positive association between dialysis incidence and the number of nephrologists per capita in adults---states with a higher number of nephrologists per 100,000 population had more incident dialysis patients per 100,000 population. Interestingly, this positive correlation is completely attenuated with adjustment for state population characteristics or patient characteristics at incident ESRD. It appears that the factor most responsible for the attenuation is percentage of African Americans in the state population or percentage of African Americans initiated on dialysis in each state (which are highly correlated r = 0.98, p < 0.001). Reflecting the well-known fact that African-American are at much higher risk of ESRD than other racial or ethnic groups in the US, the percentage of African Americans in the state population is in turn highly correlated with incident rate of ESRD per 100,000 state population in adults (r = 0.78, p < 0.001). Our interpretation of the totality of the data is that adult nephrologists relocate to states with higher burden of disease (as measured by higher ESRD incidences) and thus higher demand for their services, and those states tend to be the ones with more African-Americans, Supply may follow demand, therefore explaining our observed association, without influencing the timing of dialysis initiation.

We did not observe a similar positive correlation between the number of nephrologists per capita per state and dialysis incidence in children. It is possible that better access to chronic kidney disease care and earlier wait listing for transplantation in states with more pediatric nephrologists decreases incidence of dialysis by facilitating preemptive transplantation. Since the majority of pediatric dialysis units are affiliated with large academic centers or children’s hospitals, pediatric nephrologists may not be able to relocate as readily based on disease burden. In the case of adolescent patients, adult nephrologists at local adult dialysis units may be willing to deliver care. These factors may blunt any consistent state level correlation between physician density and dialysis incidence in our pediatric data.

Given the recent trend towards earlier dialysis initiation, along with the realization that the prevalence of CKD in the general population is quite high, concerns have been expressed regarding the adequacy of the nephrology workforce [33, 34]. Similarly, concerns have been expressed that the current pediatric nephrology workforce may not be able to meet the needs of a pediatric population that is surviving longer with CKD [35–37]. However, our data suggest that the current US workforce supply aligns with demand, at least in our adult data, and does not influence timing of dialysis initiation.

The strengths of our study include the national scope of data and the novelty of our consideration of nephrologist density as a potential influence on the timing and incidence of dialysis initiation. However, our study has limitations. Because our study is observational, causation cannot be ascribed. Workforce estimation based on AMA data may have limited accuracy and ability to capture full-time equivalency. Practitioners who have moved or failed to update their current address with AMA may be misclassified geographically, and the exact distribution of nephrologists versus patients within each state is unknown. However, defining nephrologist supply using another source of information—board certification data—did provide consistent results. Our workforce estimate cannot account for nurse practitioners and other providers who may help care for patients with kidney disease. We chose to exclude transplant patients from this study, as donor organ allocation and availability of transplant centers complicate the relationship between physician supply and demand. Furthermore, our analyses do not preclude the possibility of associations between workforce and timing of dialysis initiation on a more local level in some states or regions.

## Conclusion

In conclusion, we found no evidence that timing of dialysis initiation is influenced by supply of nephrologists. There is alignment of nephrology workforce and dialysis incidence in adults. In pediatrics, there is no significant correlation between nephrology workforce at the state level and timing of dialysis initiation or dialysis incidence, possibly due to an overall low prevalence of chronic kidney disease in children. Further studies are needed to determine the impact of any workforce shortage in nephrology and factors that contribute to the observed phenomenon of earlier dialysis initiation.

## Abbreviations

USRDS: United States Renal Data System
eGFR: Estimated glomerular filtration rate
CKD: Chronic kidney disease
ESRD: End-stage renal disease
HMO: Health maintenance organization
AMA: American Medical Association
PPD: Physician Professional Data
CHF: Congestive heart failure
ABIM: American Board of Internal Medicine
ABP: American Board of Pediatrics.
